# Albuminuria and kidney function as prognostic marker of left ventricular mass among South Asians with hypertension

**DOI:** 10.1016/j.jash.2017.10.003

**Published:** 2017-12

**Authors:** Liang Feng, Aamir Hameed Khan, Imtiaz Jehan, John Allen, Tazeen H. Jafar

**Affiliations:** a Program in Health Services & Systems Research, Duke-NUS Medical School, Singapore; b Section of Cardiology, Department of Medicine, Aga Khan University, Karachi, Pakistan; c Department of Community Health Science, Aga Khan University, Karachi, Pakistan; d Centre for Quantitative Medicine, Duke-NUS Medical School, Singapore; e Duke Global Health Institute, Duke University, Durham, NC, USA

**Keywords:** Creatinine, glomerular filtration rate, left ventricular hypertrophy

## Abstract

We aimed to evaluate the association of albuminuria and estimated glomerular filtration rate (eGFR) at baseline and changes in these parameters with left ventricular mass index (LVMI) at 7 years in adults with hypertension from communities in Pakistan. A nested cohort of 539 hypertensives aged 40 years and older from a community-living population in Karachi, Pakistan, followed up for 7 years in the Control of Blood Pressure and Risk Attenuation trial. Urine spot albumin-to-creatinine ratio (UACR) and serum creatinine-based eGFR were assessed at baseline and 7 years, and echocardiography at 7 years. Mean age of participants was 50.9 ± 9.1 (standard deviation) years; 63% were female. Mean eGFR was 91.0 ± 15.9 (standard deviation) mL/min/1.73 m^2^ and median (interquartile range) UACR 6.2 (3.9, 11.3) mg/g. In multivariate analysis, although baseline eGFR was marginally associated with LVMI, a strong association was found between higher LVMI with greater rate of decline in eGFR (β = −1.05; 95% confidence interval [CI]: [−1.94, −0.17]). Higher baseline UACR was significantly associated with higher follow-up LVMI (β = 2.26; 95% CI: [0.87, 3.65]), as was rate of UACR increase of ≥1.07 mg/g/y versus of <0.14 mg/g/y. (β = 4.19; 95% CI: [0.75, 7.63]). Associations with developing left ventricular hypertrophy were found for reduced baseline eGFR, higher baseline UACR, and greater rate of UACR increase, but not for rate of eGFR decline. Comparable results were observed for the outcomes of posterior wall thickness and septal wall thickness. Higher baseline albuminuria, lower baseline eGFR, and their longitudinal worsening were significantly associated with higher LVMI or the development of left ventricular hypertrophy among individuals with hypertension in Pakistan.

## Highlights

• Baseline albuminuria is independently associated with higher left ventricular mass (LVM).
• Rate of increase in albuminuria is independently associated with higher LVM.
• Greater rate of decline in estimated glomerular filtration rate is independently associated with higher LVM.
• Monitoring of albuminuria and estimated glomerular filtration rate could enhance cardiovascular risk stratification.

## Introduction

Higher left ventricular mass index (LVMI) or left ventricular hypertrophy (LVH) is indicative of hypertensive target organ damage and is predictive of future cardiovascular morbidity and mortality.^1^, ^2^ Kidney dysfunction, manifested as reduced estimated glomerular filtration rate (eGFR)^3^ or as albuminuria,^4^ is also a marker of hypertensive target organ damage and is independently associated with mortality and increased risk of cardiovascular event.^4^, ^5^, ^6^ In addition, LVH and kidney dysfunction often coexist, as has been shown in cross-sectional studies.^7^, ^8^

Longitudinal data are limited and suggest a bidirectional relationship between kidney dysfunction and LV mass. Higher baseline LVMI has been associated with lower eGFR and LVH with rapid decline in eGFR.^9^, ^10^ More recently, studies in individuals without advanced kidney disease showed that lower baseline eGFR and rapid decline in eGFR predicted higher future LVMI.^11^ Furthermore, baseline albuminuria and change in albuminuria have been shown to be predictive of LVH regression^12^, ^13^ and greater left ventricular mass (LVM).^14^ However, the combined impact of dynamic changes in both eGFR and albuminuria on LVM has not been reported.

The South Asian population is known to be at high risk for cardiovascular disease (CVD) and kidney disease, and related premature mortality.^15^, ^16^ However, studies in South Asians evaluating the association between kidney dysfunction and LVM are scarce, with existing reports limited largely to whites or African-American populations.^17^

We conducted a prospective study in a nested cohort of 539 individuals with hypertension from the general population in Karachi, Pakistan, with an average follow-up of 7 years, as part of the Control of Blood Pressure and Risk Attenuation (COBRA) trial.^18^ We assessed the association of baseline eGFR, change in eGFR, and baseline albuminuria and change in albuminuria, and their combined effect on LV mass after 7 years of follow-up. We hypothesized that in hypertensive adults from the general population in Karachi, reduced eGFR, and/or increased albuminuria at baseline or their worsening during follow-up, is each independently associated with higher LVM, independent of other risk factors.

## Methods

### Study Population

We conducted a post hoc analysis of data from the COBRA trial, a cluster randomized controlled trial of hypertensive individuals in communities of Karachi, Pakistan, between 2004 and 2014.^18^ Details regarding recruitment procedures have been published elsewhere.^18^ Briefly, 12 geographic census-based clusters were selected using a multistage random sampling technique. In order to examine the intervention effect, a 2 × 2 factorial design was used to randomly assign three clusters to four study groups: home health education, general practitioner training, home health education and general practitioner combined, and no intervention. Participants were chosen by door-to-door visits within each of the 12 clusters. Eligible participants were residents of selected clusters, aged 40 year and older with known hypertension or consistently elevated blood pressure (BP) on 2 of 3 visits (mean of 2 of 3 measurements of systolic BP ≥140 mm Hg or diastolic BP ≥90 mm Hg). Exclusion criteria were pregnancy or severe mental or physical disability. The Aga Khan University Ethics Review Committee granted ethical approval, and informed consent was obtained from each participant.

### Screening and Recruitment

Trained research staff visited all households in each of the 12 clusters and screened all eligible adults aged 40 years or older for hypertension after obtaining informed consent. All recruited participants underwent routine medical history taking, physical examination, and laboratory assessment. Baseline measurements were obtained in 2004–2005. A standardized questionnaire was administered to collect information on age, gender, education, smoking, self-reported antihypertensive use, history of diabetes, and history of heart disease. Body mass index (weight [kg]/height^2^ [meter]^2^) was calculated, and BP was measured three times in the sitting position. Mean values of the last two BP readings taken were used for analysis.

As previously described, serum creatinine measurements were calibrated at the Cleveland Clinic reference laboratory where serum creatinine levels were measured using the Roche enzymatic creatinine assay (in duplicate) which is traceable to the National Institute of Standards and Technology creatinine reference measurement.^19^ Glomerular filtration rate was estimated at baseline and end of study using the CKD-EPI (CKD Epidemiology Collaboration) Pakistan (CKD-EPI_PK) equation, a modified version of CKD-EPI creatinine-based equation with a correction factor (0.686 × CKD-EPI1.059) for South Asians. eGFR based on this equation denoted eGFR CKD-EPI(PK). This equation has been validated in the local population and performs better than the original CKD-EPI equation.^19^

Urine albumin excretion was measured by nephelometry using the Array Systems method on a Beckman Coulter, and creatinine (Synchron Cx-7/Delta) was measured from a morning spot urine sample. Albuminuria was evaluated by urine albumin-to-creatinine ratio (UACR).

### Follow-up at 7 Years

Trained outcomes assessors visited the homes of the participants 7 years after recruitment (2012–2014) to collect follow-up data. Informed consent was obtained for participation in the study. BP was measured, and fasting blood and urine samples were collected using the same protocol as at baseline. BP change was calculated as follow-up BP minus baseline BP. Participants were scheduled for an echocardiogram at the central health facility.

### Echocardiographic Data

The echocardiographic examination was carried out using the portable Philips CX50 imaging system by a trained sonographer using standardized procedures and rigorous quality assurance. All results were reviewed by a trained cardiologist. Left ventricular measurements were made using M-mode (MM) and two-dimensional (2D) echo from the parasternal long axis, adhering to American Society of Echocardiography guidelines.^20^ As in the LIFE Study,^21^ end-diastolic left ventricular septal and posterior wall thickness (PWT) and internal dimensions were used to calculate LVM using the formula: LVM = 1.04 × 0.8 ([left ventricular wall thicknesses + internal dimension] − [internal dimension]) + 0.6 g. LVMI was calculated as LVM (based on MM measurements) in grams divided by body surface area in square meters. Two trained sonographers performed all imaging for the study. Interrater reliability among sonographers for LVMI was very good, with kappa = 0.93.

### Analysis

The primary outcome was LVMI, and the secondary outcome was LVH defined as LVMI greater than 88 g/m^2^ in females and greater than 102 g/m^2^ in males.^22^

The ancillary outcomes of PWT at end diastole and septal wall thickness (SWT) at end diastole were also analyzed.

Besides baseline eGFR and baseline UACR, the main exposure variables were (1) rate of decline in eGFR defined as the difference between follow-up and baseline eGFR divided by study duration in years and (2) rate of increase in albuminuria defined as the difference between follow-up and baseline UACR divided by study duration in years. A categorical variable was created using UACR tertile.

### Statistical Analysis

Baseline characteristics together with rate of decline in eGFR and increase in UACR as well as change in systolic blood pressure (SBP) and diastolic blood pressure (DBP) were summarized as mean and standard deviation or median and interquartile range for continuous variables and as counts and percentage for categorical variables. Comparisons between groups with and without LVH were conducted using the 2-sample t-test for normally distributed continuous variables and a chi-square test for categorical variables. The Mann–Whitney U test was used to compare groups for nonnormal continuous variables. The bivariate correlations between SBP change and kidney biomarkers were evaluated using Pearson correlation coefficient or analysis of variance, where appropriate.

Associations between follow-up LVMI and the markers eGFR and UACR unadjusted and adjusted for potential confounders were investigated using linear regression analysis. Six models were developed by systematically selecting variables from a candidate set and introducing them into the models:

• Model 1: Baseline eGFR; baseline UACR (model 1 for each variable)
• Model 2: Baseline eGFR + baseline UACR
• Model 3: Variables in model 2 + age, gender, education, BMI, diabetes, heart disease, SBP, DBP, smoking, low-density lipoproteins (LDLs), high-density lipoproteins, triglyceride, angiotensin-converting enzyme inhibitor or angiotensin II receptor blocker use, calcium channel blocker use, β-blocker use, diuretics use, and intervention group.
• Model 4: Variables in model 3 + SBP change, DBP change, and rate of decline in eGFR
• Model 5: Variables in model 3 + SBP change, DBP change, and rate of increase in UACR
• Model 6: Variables in model 3 + SBP change, DBP change, rate of increase in UACR, and rate of decline in eGFR

Using the same model building approach, we performed logistic regression to determine whether the two markers were independently associated with follow-up LVH. In addition, both PWT and SWT were analyzed in model 6. We log-transformed UACR in all analyses given its right-skewed distribution. We explored interaction effects of kidney biomarkers with age, gender, SBP change, and DBP change in multiple linear regression analysis. We also conducted a sensitivity analysis on model 6 by restricting the analysis to individuals without heart disease. All analyses accounted for clustering by household at the census level as a random effect using the SAS GLIMMIX procedure. Sensitivity analyses were performed using 2D measurements for LVMI. A two-sided *P* value of .05 was considered statistically significant. Statistical analyses were performed using SAS, version 9.4 (SAS Institute, Inc, Cary, NC).

## Results

### Study Population

Of all the original 1341 trial participants, 311 individuals died, 198 migrated, and 92 were lost to follow-up, resulting in 740 in the 7-year follow-up posttrial follow-up study. Of the 740, 169 (22.8%) did not undergo echocardiograms for either refusals or failure to keep appointment, and 571 had echocardiographic data. From among the latter, we excluded 26 (1.9%) with no baseline or follow-up serum creatinine, urine albumin or urine creatinine, three missing parameters for LVMI, three missing information on heart disease, 10 missing LDL, high-density lipoprotein, and triglyceride, as well as one missing follow-up DBP, leaving 539 (40% of the original cohort and 73% of those at 7-year follow-up) individuals for analysis.

Compared to the excluded individuals from the original cohort (n = 802), those analyzed (n = 539) were younger and had higher education levels and lower rates of diabetes, heart disease, and smoking. They also had higher baseline BMI and eGFR, but lower baseline SBP and baseline UACR (Table S1).

The mean age among the 539 hypertensives included in the analysis was 50.9 ± 9.1 years, 63% were female and 34% had no formal education (Table 1). Mean LVMI was 69.9 ± 17.5 g/m^2^, mean eGFR 91.0 ± 15.9 mL/min/1.73 m^2^, and median UACR (interquartile range) 6.2 (3.9, 11.3) mg/g.

**Table 1:** Baseline characteristics by left ventricular hypertrophy (LVH) among the hypertensive participants (n = 539)

Variables	Total	Male			Female		
		No LVH (n = 185)	LVH (n = 14)	*P* Value	No LVH (n = 298)	LVH (n = 42)	*P* Value
Age, mean (SD)	50.9 (9.1)	51.2 (8.5)	53.4 (10.6)	.37	49.9 (9.0)	56.8 (9.2)	<.001
Education, n (%)				1.00			.78
Formal	358 (66.4)	161 (87.0)	12 (85.7)		163 (54.7)	22 (52.4)	
Not formal	181 (33.6)	24 (13.0)	2 (14.3)		135 (45.3)	20 (47.6)	
Waist circumference (cm)	93.1 (11.0)	96.1 (10.2)	92.7 (15.0)	.42	91.0 (10.9)	94.6 (10.9)	.043
SBP (mm Hg, mean, SD)	149.1 (22.6)	149.4 (20.7)	142.9 (16.8)	.25	147.8 (23.6)	158.8 (22.9)	.005
DBP (mm Hg, mean, SD)	92.9 (12.3)	93.4 (12.4)	89.6 (13.5)	.26	92.7 (12.3)	92.9 (11.0)	.89
Antihypertensive use, n (%)	205 (38.0)	62 (33.5)	2 (14.3)	.23	119 (39.9)	22 (52.4)	.13
ARB or ACEI, n (%)	71 (13.2)	24 (13.0)	0 (0.0)	.23	39 (13.1)	8 (19.1)	.29
β-Blocker, n (%)	119 (22.1)	39 (21.1)	2 (14.3)	.74	64 (21.5)	14 (33.3)	.087
CCB, n (%)	51 (9.5)	13 (7.0)	1 (7.1)	1.00	33 (11.1)	4 (9.5)	1.00
Diuretics, n (%)	12 (2.2)	3 (1.6)	0 (0.0)	1.00	7 (2.4)	2 (4.8)	.31
Diabetes, n (%)	129 (23.9)	32 (17.3)	5 (35.7)	.14	80 (26.9)	12 (28.6)	.81
Heart disease, n (%)	64 (11.9)	21 (11.4)	2 (14.3)	.67	38 (12.8)	3 (7.1)	.30
Smoking, n (%)				.10			.81
Never	333 (61.8)	82 (44.3)	3 (21.4)		218 (73.2)	30 (71.4)	
Current or past	206 (38.2)	103 (55.7)	11 (78.6)		80 (26.9)	12 (28.6)	
LDL (mg/dL, mean, SD)	120.4 (32.9)	118.1 (31.9)	100.2 (25.2)	.042	122.0 (34.0)	126.3 (29.0)	.44
HDL (mg/dL, mean, SD)	40.3 (10.3)	36.3 (7.3)	39.0 (8.6)	.19	42.7 (11.1)	41.6 (11.1)	.55
Triglyceride (mg/dL, mean, SD)	179.4 (102.0)	192.8 (125.8)	191.5 (130.9)	.68	168.8 (80.6)	191.9 (105.7)	.42
eGFR (mL/min/1.73 m^2^, mean, SD)	91.0 (15.9)	86.4 (14.9)	75.4 (26.8)	.12	95.5 (13.5)	84.3 (19.4)	<.001
UACR (mg/g, median, IQR)	6.2 (3.9, 11.3)	4.9 (3.1, 8.1)	8.2 (3.7, 13.8)	.18	6.6 (4.3, 11.8)	9.6 (6.1, 29.3)	.003
Intervention group, n (%)				.17			.79
No intervention	137 (25.4)	49 (26.5)	3 (21.4)		77 (25.8)	8 (19.1)	
GP only	107 (19.9)	40 (21.6)	6 (42.9)		53 (17.8)	8 (19.1)	
HHE only	129 (23.9)	44 (23.8)	4 (28.6)		71 (23.8)	10 (23.8)	
GP and HHE	166 (30.8)	52 (28.1)	1 (7.1)		97 (32.6)	16 (38.1)	

ACEI, angiotensin-converting enzyme inhibitor; ARB, angiotensin II receptor blocker; CCB, calcium channel blocker; DBP, diastolic blood pressure; eGFR, estimated glomerular filtration rate; GP, general practitioner; HDL, high-density lipoprotein; HHE, home health education; IQR, interquartile range; LDL, low-density lipoprotein; SBP, systolic blood pressure; SD, standard deviation; UACR, urine albumin to creatinine ratio.

Mann–Whitney U test for UACR, LVH was defined as LVMI > 88 g/m^2^ for women, >102 g/m^2^ for man.

Among men, participants with LVH had lower levels of LDL, higher SBP change, and higher rate of UACR increase than those without LVH. Among women, participants with LVH were characterized by older age, higher waist circumference, higher baseline SBP, lower baseline eGFR, higher baseline UACR, higher SBP increase, and higher rate of UACR increase than those without LVH (Table 1, Table 2). There were significant bivariate associations of change in SBP over 7 years with baseline UACR (r = −0.12, *P* = .005), rate of UACR increase (F_2,536_ = 3.46, *P* = .032), and rate of eGFR decline (r = 0.12, *P* = .007), each.

### Left Ventricular Mass Index

Table 3 shows the association of baseline, rate of decline in eGFR, and rate of increase in UACR with LVMI at 7 years. In the univariate model (model 1), each 1 mL/min/1.73 m^2^ decrease in baseline eGFR was significantly associated with a 0.21 g/m^2^ increase in LVMI, and per unit increase in log-transformed UACR (about 2.7 times greater in UACR) was predictive of 2.87 g/m^2^ higher LVMI. These associations were attenuated but remained statistically significant when both markers (baseline eGFR and baseline UACR) were introduced together in model 2. With further adjustment for demographic variables and other confounders in models 3, 4, and 5, statistical significance persisted only for baseline UACR. Rate of decline in eGFR and increase in UACR were significantly associated with LVMI in models 4 and 5. After adjustment for rate of decline in eGFR and increase in UACR in model 6, the significant association between baseline UACR and LVMI persisted. In addition, every 1 mL/min/1.73 m^2^/y decline in eGFR was significantly associated with 1.05 g/m^2^ higher LVMI at the end of follow-up (*P* = .02). LVMI significantly increased by 4.19 g/m^2^ in those with rate of UACR increase of ≥1.07 mg/g/y compared to individuals with UACR rate of increase of <0.14 mg/g/y (*P* = .02), and although not statistically significant, by 2.65 g/m^2^ in those with rate of UACR increase of between 0.14 and 1.07 mg/g/y (*P* = .15). Other covariates positively associated with LVMI were waist circumference (β = 0.18; 95% confidence interval [CI]: [0.04, 0.32]), SBP (β = 0.29; 95% CI: [0.18, 0.40]), and SBP change (β = 0.25; 95% CI: [0.15, 0.35]). No significant interactions were observed between age, gender, change in SBP, or change in DBP with kidney biomarkers on LVMI. Analysis results remained unchanged after excluding individuals with heart disease at baseline (Table S2).

**Table 2:** Change in blood pressure and renal function by left ventricular hypertrophy (LVH) among the hypertensive participants (n = 539)∗

Variables	Total	Male			Female		
		No LVH (n = 185)	LVH (n = 14)	*P* Value	No LVH (n = 298)	LVH (n = 42)	*P* Value
SBP change (mm Hg)				.032			.026
Mean, SD	−0.1 (24.1)	0.8 (21.1)	13.8 (29.0)		−2.3 (24.9)	6.9 (26.9)	
Median, IQR	2 (−15.5, 15.5)	2.0 (−11.0, 15.0)	8.8 (−5.0, 32.0)		0 (−17.5, 13.5)	9.3 (−6.0, 20.5)	
DBP change (mm Hg)				.081			.53
Mean, SD	−6.3 (14.2)	−5.7 (13.0)	0.7 (14.8)		−7.1 (14.7)	−5.6 (15.6)	
Median, IQR	−6.0 (−14.0, 3.5)	−5.0 (−14.0, 3.5)	−0.5 (−15.0, 13.5)		−6.0 (−15.0, 2.0)	−4.0 (−14.0, 7.0)	
Rate of decline in eGFR (mL/min/1.73 m^2^/y)†				.15			.38
Mean, SD	−0.3 (1.8)	0.2 (1.3)	−0.3 (1.8)		−0.4 (1.9)	−0.8 (2.4)	
Median, IQR	0.0 (−0.8, 0.7)	0.1 (−0.6, 0.8)	0.1 (−0.7, 1.4)		0.0 (−1.3, 0.7)	0 (−2.7, 0.7)	
Rate of increase in UACR (mg/g/y, median, IQR)‡	0.5 (0.0, 1.6)	0.3 (0.0, 1.5)	1.3 (0.4, 5.0)	.040	0.5 (0.0, 1.5)	1.2 (0.1, 4.1)	.045
Rate of increase in UACR (mg/g/y)				.16			.01
<0.14	179 (33.2)	71 (38.4)	2 (14.3)		96 (32.2)	10 (23.8)	
0.14–1.07	178 (33.0)	55 (29.7)	5 (35.7)		109 (36.6)	9 (21.4)	
≥1.07	182 (33.8)	59 (31.9)	7 (50.0)		93 (31.2)	23 (54.8)	

DBP, diastolic blood pressure; eGFR, estimated glomerular filtration rate; IQR, interquartile range; LVMI, left ventricular mass index; SBP, systolic blood pressure; SD, standard deviation; UACR, urine albumin-to-creatinine ratio.

Mann–Whitney U test for rate of increase in UACR, LVH was defined as LVMI > 88 g/m^2^ for women, >102 g/m^2^ for men.

∗ Change in blood pressure was calculated using blood pressure at year 7 minus baseline blood pressure.

† Rate of decline in eGFR = (eGFR at year 7 − eGFR at baseline)/duration of follow up (in y).

‡ Rate of increase in UACR = (UACR at year 7 − UACR at baseline)/duration of follow up (in y).

**Table 3:** Association of baseline and rate of increase in eGFR (mL/min/1.73 m^2^) and UACR (mg/g) with LVMI (g/m^2^) (n = 539) from multiple regression analysis

Variables	Model 1		Model 2		Model 3		Model 4		Model 5		Model 6	
	β-Coefficient (95% CI)	*P* Value	β-Coefficient (95% CI)	*P* Value	β-Coefficient (95% CI)	*P* Value	β-Coefficient (95% CI)	*P* Value	β-Coefficient (95% CI)	*P* Value	β-Coefficient (95% CI)	*P* Value
Baseline eGFR	−0.21 (−0.30, −0.12)	<.001	−0.18 (−0.28, −0.09)	<.001	−0.07 (−0.19, 0.05)	.23	−0.11 (−0.23, 0.01)	.06	−0.08 (−0.19, 0.04)	.18	−0.11 (−0.23, 0.00)	.06
Baseline UACR	2.87 (1.55, 4.18)	<.001	2.51 (1.20, 3.82)	<.001	2.08 (0.72, 3.45)	.003	2.00 (0.68, 3.32)	.003	2.31 (0.92, 3.72)	.001	2.26 (0.87, 3.65)	.002
Gender (female)					−1.35 (−5.03, 2.34)	.47	−1.02 (−4.57, 2.54)	.57	−0.93 (−4.49, 2.63)	.61	−1.23 (−4.78, 2.33)	.50
Waist circumference					0.16 (0.02, 0.30)	.027	0.18 (0.05, 0.32)	.009	0.18 (0.04, 0.31)	.01	0.18 (0.04, 0.32)	.01
Baseline SBP					0.16 (0.06, 0.27)	.003	0.30 (0.18, 0.41)	<.001	0.29 (0.18, 0.40)	<.001	0.29 (0.18, 0.40)	<.001
SBP change∗							0.26 (0.16, 0.35)	<.001	0.24 (0.14, 0.34)	<.001	0.25 (0.15, 0.35)	<.001
Rate of decline in eGFR†							−1.15 (−2.03, −0.28)	.010			−1.05 (−1.94, −0.17)	.02
Rate of increase in UACR‡										.03		.04
<0.14 (Ref)									1.00		1.00	
0.14–1.07									2.56 (−1.07, 6.19)	.17	2.65 (−0.97, 6.27)	.15
≥1.07									4.61 (1.18, 8.05)	.009	4.19 (0.75, 7.63)	.02

ACEI, angiotensin-converting enzyme inhibitor; ARB, angiotensin II receptor blocker; CCB, calcium channel blocker; CI, confidence interval; DBP, diastolic blood pressure; eGFR, estimated glomerular filtration rate; HDL, high-density lipoprotein; LDL, low-density lipoprotein; LVMI, left ventricular mass index; SBP, systolic blood pressure; UACR, urine albumin-to-creatinine ratio.

Model 1: Baseline eGFR, baseline UACR (model 1 for each variable).

Model 2: Baseline eGFR + baseline UACR.

Model 3: Variables in model 2 + age, gender, education, waist circumference, diabetes, heart disease, SBP, DBP, smoking, LDL, HDL, triglyceride, ACEI or ARB use, CCB use, β-blocker use, diuretics use, and intervention group.

Model 4: Variables in model 3 + SBP change, DBP change, and rate of decline in eGFR.

Model 5: Variables in model 3 + SBP change, DBP change, and rate of increase in UACR.

Model 6: Variables in model 3 + SBP change, DBP change, rate of increase in UACR, and rate of decline in eGFR.

All models accounted for clustering effect by household as a random effect.

In addition to kidney function biomarkers and gender, variables with *P* value < .05 in model 6 were reported in the table.

∗ Change in SBP was calculated using SBP at year 7 minus baseline SBP.

† Rate of decline in eGFR = (eGFR at year 7 − eGFR at baseline)/duration of follow-up (in y).

‡ Rate of increase in UACR = (UACR at year 7 − UACR at baseline)/duration of follow-up (in y).

Sensitivity analysis using 2D-mode measurement of LVMI yielded consistent results.

### Left Ventricular Hypertrophy

The adjusted associations between kidney biomarkers and LVH are summarized in Table 4. In the final model (model 6), both baseline eGFR and baseline UACR were significantly associated with LVH (eGFR: odds ratio [OR] = 0.97; 95% CI: [0.95, 0.99]; UACR: OR = 1.37; 95% CI: [1.04, 1.80]). Rate of UACR increase group of ≥1.07 mg/g/y conferred a 2.17-time greater risk of LVH (OR = 2.17; 95% CI: [0.97–4.83]) using <0.14 mg/g/y as the reference group. In contrast, there was no association between rate of eGFR decline and LVH, albeit the direction of association was similar to that of LVMI.

**Table 4:** Association of baseline and rate of increase in eGFR (mL/min/1.73 m^2^) and UACR (mg/g) with LVH from logistic regression analysis (n = 539)

Variables	Model 1		Model 2		Model 3		Model 4		Model 5		Model 6	
	OR (95% CI)	*P* Value	OR (95% CI)	*P* Value	OR (95% CI)	*P* Value	OR (95% CI)	*P* Value	OR (95% CI)	*P* Value	OR (95% CI)	*P* Value
Baseline eGFR	0.97 (0.95, 0.98)	<.001	0.97 (0.96, 0.99)	<.001	0.97 (0.95, 0.99)	.01	0.97 (0.95, 0.99)	.004	0.97 (0.95, 0.99)	.01	0.97 (0.95, 0.99)	.006
Baseline UACR	1.44 (1.17, 1.76)	<.001	1.32 (1.06, 1.63)	.01	1.28 (1.01, 1.63)	.04	1.30 (1.01, 1.67)	.04	1.39 (1.05, 1.83)	.02	1.37 (1.04, 1.80)	.03
Gender (female)					3.09 (1.34, 7.14)	.008	3.43 (1.42, 8.31)	.006	3.45 (1.43, 8.32)	.006	3.29 (1.36, 7.96)	.008
Baseline SBP					1.01 (0.99-1.04)	.18	1.03 (1.01, 1.06)	.006	1.03 (1.01, 1.06)	.01	1.03 (1.01, 1.06)	.01
SBP change∗							1.03 (1.01, 1.05)	.001	1.03 (1.01, 1.05)	.004	1.03 (1.01, 1.05)	.003
Rate of decline in eGFR†							0.87 (0.73, 1.02)	.10			0.89 (0.75, 1.06)	.19
Rate of increase in UACR‡										.11		.17
<0.14									1.00		1.00	
0.14–1.07									1.65 (0.64, 4.25)	.30	1.64 (0.64, 4.23)	.31
≥1.07									2.33 (1.06, 5.14)	.04	2.17 (0.97, 4.83)	.06

ACEI, angiotensin-converting enzyme inhibitor; ARB, angiotensin II receptor blocker; CCB, calcium channel blocker; CI, confidence interval; DBP, diastolic blood pressure; eGFR, estimated glomerular filtration rate; HDL, high-density lipoprotein; LDL, low-density lipoprotein; LVH, left ventricular hypertrophy; OR, odds ratio; SBP, systolic blood pressure; UACR, urine albumin-to-creatinine ratio.

Model 1: Baseline eGFR; baseline UACR (model 1 for each variable).

Model 2: Baseline eGFR + baseline UACR.

Model 3: Variables in model 2 + age, gender, education, waist circumference, diabetes, heart disease, SBP, DBP, smoking, LDL, HDL, triglyceride, ACEI or ARB use, CCB use, β-blocker use, diuretics use, and intervention group.

Model 4: Variables in model 3 + SBP change, DBP change, and rate of decline in eGFR.

Model 5: Variables in model 3 + SBP change, DBP change, and rate of increase in UACR.

Model 6: Variables in model 3 + SBP change, DBP change, rate of increase in UACR, and rate of decline in eGFR.

All models accounted for clustering effect by household as a random effect.

In addition to kidney function biomarkers, variables with *P* value < .05 in model 6 were reported.

∗ Change in SBP was calculated using SBP at year 7 minus baseline SBP.

† Rate of decline in eGFR = (eGFR at year 7 − eGFR at baseline)/duration of follow-up (in y).

‡ Rate of increase in UACR = (UACR at year 7 − UACR at baseline)/duration of follow-up (in y).

### PWT and SWT

Table 5 shows the multivariate association of kidney biomarkers with the outcomes of PWT and SWT. Higher baseline UACR and greater rate of eGFR decline were significantly associated with increase in PWT. Rate of UACR increase between groups of 0.14 and 1.07 mg/g/y and ≥1.07 mg/g/y both was associated with higher PWT as compared with group of rate <0.14 mg/g/y. However, baseline eGFR had no association with PWT. As for SWT, both baseline UACR and rate of increase in UACR were positively associated with SWT. In contrast, no association was identified between baseline eGFR or its rate of decline and SWT. Sensitivity analysis using 2D-mode measurements of PWT and SWT yielded consistent results.

**Table 5:** Association of baseline and rate of increase in eGFR (mL/min/1.73 m^2^) and UACR (mg/g) with posterior wall thickness (cm) and septal wall thickness (cm) (n = 539) from multiple regression analysis

Variables	Posterior Wall Thickness (M Mode)		Septal Wall Thickness (M Mode)	
	β Coefficient (95% CI)	*P* Value	β Coefficient (95% CI)	*P* Value
Baseline eGFR	0.0001 (−0.0007, 0.0008)	.83	0.0003 (−0.0005, 0.0012)	.44
Baseline UACR	0.0114 (0.0025, 0.0203)	.013	0.0120 (0.0021, 0.0219)	.018
Rate of decline in eGFR∗	−0.0075 (−0.0132, −0.0019)	.009	−0.0010 (−0.0072, 0.0053)	.76
Rate of increase in UACR†		.048		.019
<0.14 (Ref)				
0.14–1.07	0.0260 (0.0028, 0.0492)	.028	0.0285 (0.0028, 0.0542)	.03
≥1.07	0.0232 (0.0011,0.0452)	.039	0.0329 (0.0085, 0.0573)	.008
Gender (female)	−0.0320 (−0.0548, −0.0092)	.006	−0.0362 (−0.0614, −0.0109)	.005
Waist circumference	0.0024 (0.0015, 0.0032)	<.001	0.0022 (0.0013, 0.0032)	<.001
Baseline SBP	0.0014 (0.0006, 0.0021)	<.001	0.0015 (0.0007, 0.0023)	<.001
SBP change‡	0.0007 (0.0001, 0.0013)	.022	0.0006 (−0.0001, 0.0013)	.098

ARB, angiotensin II receptor blocker; ACEI, angiotensin-converting enzyme inhibitor; CCB, calcium channel blocker; DBP, diastolic blood pressure; eGFR, estimated glomerular filtration rate; HDL, high-density lipoprotein; LDL, low-density lipoprotein; SBP, systolic blood pressure; UACR, urine albumin-to-creatinine ratio.

Note: Variables in the model were baseline eGFR, baseline UACR, rate of decline in eGFR, rate of increase in UACR, age, gender, education, waist circumference, diabetes, heart disease, SBP, SBP change, DBP, DBP change, smoking, LDL, HDL, triglyceride, ACEI or ARB use, CCB use, β-blocker use, diuretics use, and intervention group.

All models accounted for clustering effect by household as a random effect.

In addition to kidney function biomarkers, variables with *P* value < .05 for either outcomes were reported in the table.

∗ Rate of decline in eGFR = (eGFR at year 7 − eGFR at baseline)/duration of follow-up (in y).

† Rate of increase in UACR = (UACR at year 7 − UACR at baseline)/duration of follow-up (in y).

‡ Change in SBP was calculated using SBP at year 7 minus baseline SBP.

## Discussion

Our study examining the association of markers of kidney function and damage with LVMI among 539 hypertensive individuals with near normal kidney function from the general population in communities in Pakistan found that higher baseline UACR, greater rate of increase in UACR, and greater rate of decline in eGFR were strongly and independently associated with higher LVMI at the 7-year follow-up. Similar results were found for PWT and SWT, except that rate of eGFR decline was not associated SWT. Higher baseline UACR, lower baseline eGFR, and the rate of increase in UACR of ≥1.07 mg/g/y versus <0.14 mg/g/y predicted greater risk of LVH. Our findings based on the first study of its kind in communities in South Asia have tremendous clinical and public health implications for screening and monitoring kidney markers, each offering prognostic information for risk stratification of individuals at high risk of adverse cardiac outcomes, especially in South Asians—a population known to be at high risk of CVD.^15^, ^16^

Our findings corroborate studies in other populations^9^, ^10^, ^11^, ^12^, ^13^ indicating that kidney dysfunction as a static measure or evidence of progressive worsening over time predicted higher LVMI. Presence of albuminuria has been shown to predict future LVMI in individuals with hypertension.^23^, ^24^ A reduction in microalbuminuria has been shown to be a significant predictor of reduced chance of LVH regression.^12^ In addition, in a report by Bansal et al,^11^ decline in eGFR was significantly associated with higher LVMI 10 years later in a sample of 2410 black and white participants with baseline eGFR >60 mL/min/1.73 m^2^. Furthermore, parallel worsening of eGFR and albuminuria, compared to either alone, has been associated with greater LVMI.^24^ Our study extends previous research by evaluating dynamic change in UACR and eGFR in combination for prediction of cardiac abnormalities and to demonstrate independent long-term effects of markers of both static and dynamic kidney damage parameters on LVMI in hypertensive individuals in low-income communities in South Asia.

It is important to highlight that in our study, change in SBP was significantly correlated with baseline UACR, rate of UACR increase, rate of eGFR decline, and LVMI. However, the associations between markers of kidney damage with LVMI were independent of baseline SBP and its change over the follow-up. Few studies adjusted for change in SBP when examining the effect of kidney dysfunction on cardiac structure.^11^, ^13^

It is also interesting to note that decline in eGFR was associated with increase in PWT; however, its relationship with SWT was not significant. This suggests the possibility of eccentric left ventricular hypertrophy being associated with reduced kidney function which has been reported previously.^25^

The mechanisms linking kidney dysfunction to cardiac abnormalities are still unclear. Traditional cardiovascular risk factors at baseline such as hypertension, diabetes, high cholesterol, and smoking do not entirely explain the associations because they were controlled for in the analysis. Likewise, the multivariable analysis was adjusted for greater decline in systolic BP among those without LVH compared to those with LVH. In patients with chronic kidney disease, anemia is a potential factor mediating the association between markers of kidney dysfunction and LVMI. Average eGFR was high in our population (91 mL/min/1.73 m^2^) with only two subjects having an eGFR of <30 mL/min/1.73 m^2^. Therefore, we believe that low hemoglobin may not play a major role in the observed associations. Albuminuria is theoretically related to multiple pathophysiological processes including comorbidities, systemic inflammation, and endothelial dysfunction and is a marker of generalized cardiovascular damage.^26^, ^27^ Other possible factors could be overactivity of the renin–aldosterone system and sympathetic tone and alteration in mineral metabolism such as 1,25-hydroxyvitamin D, parathyroid hormone, and fibroblast growth factor 23,^28^, ^29^, ^30^ all of which are associated with progressive kidney dysfunction and contribute to the development and worsening of cardiac damage.

There are limitations in the study. First, echocardiography was not performed at baseline, and we were not able to evaluate possible confounding by baseline LVMI. However, we accounted for the presence of known heart disease in the main analysis. Further, sensitivity analysis after exclusion of individuals with heart disease yielded consistent results. Nevertheless, residual confounding by subclinical structural change could not be controlled for adequately. For this reason, it is not possible to establish a cause-effect relationship in this study. However, our findings do establish the predictive association of eGFR and UACR with LVMI and LVH. Second, 40% of the original COBRA cohort were available with echocardiographic data for analysis at 7-year follow-up. The analytic sample represents relatively healthier hypertensive individuals with better kidney function and lower prevalence of heart disease and diabetes than the general population in the urban communities of Pakistan. Consequently, we expect higher associations would be found in individuals with more adverse risk factor profile. However, studies elsewhere have reported potential utility of eGFR and UACR to the Framingham risk score as prognostic of CVD mortality in patients with advanced kidney disease.^31^ Thus, our findings might be generalizable to most of the general population in the communities in South Asia and possibly neighboring countries, but additional studies are required in more representative samples of hypertensive individuals with a wide spectrum risk profile. Third, history of hypertension and legacy effect of uncontrolled BP were not accounted for in the analysis. However, use of antihypertensive medications is known to lead to LVH regression.^32^ Fourth, we only measured kidney biomarkers at baseline and end of follow-up and thus can only examined the linear changes in kidney biomarkers over time. Finally, LVMI measurements using MM and 2D echocardiographic imaging have limitations including operator-dependent technical issues such as image quality and beam positioning as well as the assumption of a uniform geometric shape of the left ventricle. However, the sonographers performing imaging in our study were trained as per ASE guidelines with good interrater reproducibility measures.^33^

The main strengths of our study include a community-based sample identified using door-to-door census in South Asia and rigorous measurement procedures enhancing generalizability of findings to public health settings. Other major strengths include long follow-up duration, improved accuracy of glomerular filtration rate estimate using a locally modified CKD-EPI creatinine equation and IDMS calibrated serum creatinine, adjustment for important confounders rarely considered in early studies (eg, BP change and lipid profile), and high quality echocardiographic imaging with sound reliability measurements. In addition, results of sensitivity analysis using 2D imaging were consistent with MM, as were ancillary analyses for association of all kidney biomarkers with PWT and UACR with SWT. Thus, we believe our findings are robust.

Our findings have tremendous implications for public health and clinical practice especially in South Asian countries where CVD is the leading cause of mortality accounting for one-third of all deaths with tremendous economic consequences.^15^

Point of care testing for kidney function is available across a variety of primary care settings, including care by trained health workers providing home health checks and education in resource-constrained regions globally.^34^ The PREVEND Study in the Netherlands demonstrated that lowering albuminuria can prevent heart failure.^35^ Our results suggest that screening for both eGFR and UACR at baseline and during follow-up should be evaluated for risk stratification and subsequent prevention of future LVH and CVD in South Asians with hypertension. Such an approach is likely to be cost-effective especially in resource-constrained settings where access to cardiac imaging technologies is limited.

In conclusion, higher baseline albuminuria, lower baseline eGFR, and their longitudinal worsening over 7 years were significantly associated with higher LVMI or the development of LVH among individuals with hypertension in Pakistan. The findings suggest that both baseline screening and follow-up monitoring eGFR and UACR could potentially enhance cardiovascular risk stratification for cardiac structural damage and subsequent CVD. Future studies should consider both static and dynamic marker of kidney dysfunction for risk stratification and prevention of CVD.

## Figures and Tables

**Table S1:** Comparison of baseline characteristics between individuals in analytic sample and those excluded from the analysis

Variable	n	Analytic Sample (n = 539)	Excluded (n = 802)	*P* Value
Age, mean (SD)		50.9 (9.06)	55.9 (12.2)	<.001
Female, n (%)		340 (63.1)	500 (62.3)	.78
Education, n (%)				<.001
Formal		358 (66.2)	429 (53.5)	
Not formal		181 (33.6)	373 (46.5)	
BMI (kg/m^2^, mean, SD)	1337	27.7 (5.0)	26.0 (5.5)	<.001
SBP (mm Hg, mean, SD)		149.1 (22.6)	153.2 (25.6)	.002
DBP (mm Hg, mean, SD)		92.9 (12.3)	93.1 (13.6)	.78
ARB or ACEI, n (%)		71 (13.2)	112 (14.0)	.68
β-Blocker, n (%)		119 (22.1)	153 (19.1)	.18
CCB, n (%)		51 (9.5)	78 (9.7)	.87
Diuretics, n (%)		12 (2.2)	27 (3.4)	.22
Diabetes, n (%)	1279	129 (23.9)	241 (32.6)	<.001
Heart disease, n (%)	1334	64 (11.9)	119 (14.8)	.11
Smoking, n (%)				<.001
Never		333 (61.8)	401 (50.0)	
Current or past		206 (38.2)	401 (50.0)	
LDL (mg/dL, mean, SD)	1271	120.4 (32.9)	118.9 (32.4)	.40
HDL (mg/dL, mean, SD)	1271	40.3 (10.3)	40.9 (10.7)	.31
Triglyceride (mg/dL, mean, SD)	1271	179.4 (102)	165 (84.1)	.007
eGFR (mL/min/1.73 m^2^, mean, SD)	1271	91.0 (15.9)	84.2 (20.9)	<.001
UACR (mg/g, median, IQR)	1272	6.2 (3.9, 11.3)	6.9 (4.4, 19.7)	<.001
Intervention group, n (%)				<.001
No intervention		137 (25.4)	189 (23.6)	
GP only		107 (19.9)	228 (28.4)	
HHE only		129 (23.9)	219 (27.3)	
GP and HHE		166 (30.8)	166 (20.7)	

ACEI, angiotensin-converting enzyme inhibitor; ARB, angiotensin II receptor blocker; BMI, body mass index; CCB, calcium channel blocker; DBP, diastolic blood pressure; eGFR, estimated glomerular filtration rate; GP, general practitioner; HDL, high-density lipoprotein; HHE, home health education; IQR, interquartile range; LDL, low-density lipoprotein; SBP, systolic blood pressure; SD, standard deviation; UACR, urine albumin-to-creatinine ratio.

**Table S2:** Association of baseline and rate of increase in eGFR (mL/min/1.73 m^2^) and UACR (mg/g) with LVMI (g/m^2^) in individuals without heart disease (n = 475) from multiple regression

Variables	Without Heart Disease N = 475	
	β-Coefficient 95% (CI)	*P* Value
Baseline eGFR	−0.12 (−0.24, 0.01)	.06
Baseline UACR	2.29 (0.80, 3.77)	.003
Gender (female)	−1.96 (−5.90, 1.98)	.33
BMI	0.31 (−0.02, 0.65)	.06
Baseline SBP	0.30 (0.18, 0.42)	<.001
SBP change∗	0.26 (0.16, 0.36)	<.001
Rate of increase in eGFR†	−1.04 (−2.00, −0.09)	.03
Rate of increase in UACR‡		.05
<0.14	Reference	
0.14–1.07	2.86 (−1.05, 6.77)	.15
≥1.07	4.60 (0.87, 8.33)	.02

BMI, body mass index; CI, confidence interval; eGFR, estimated glomerular filtration rate; LVMI, left ventricular mass index; SBP, systolic blood pressure; UACR, urine albumin-to-creatinine ratio.

Analysis was based on model 6 including all covariates and accounted for clustering effect by household as a random effect.

∗ Change in SBP was calculated using SBP at year 7 minus baseline SBP.

† Rate of increase in eGFR = (eGFR at year 7 − eGFR at baseline)/duration of follow-up (in y).

‡ Rate of increase in UACR = (UACR at year 7 − UACR at baseline)/duration of follow-up (in y).
